# The complete mitochondrial genome of *Electrona carlsbergi* (Myctophiformes, Myctophidae) with phylogenetic consideration

**DOI:** 10.1080/23802359.2017.1383200

**Published:** 2018-02-01

**Authors:** Shuzhang Liang, Wei Song, Chunyan Ma, Fengying Zhang, Keji Jiang, Luming Wang, Lingbo Ma

**Affiliations:** a Key Laboratory of Oceanic and Polar Fisheries, Ministry of Agriculture, East China Sea Fisheries Research Institute, Chinese Academy of Fishery Sciences, Shanghai, China;; b College of Fisheries and Life Sciences, Shanghai Ocean University, Shanghai, China

**Keywords:** *Electrona carlsbergi*, mito-genome, phylogenetic position, phylogenetic tree

## Abstract

The complete mitochondrial genome of *Electrona carlsbergi* was obtained, which was 18,282 bp in size and including 13 protein-coding genes, 2 ribosomal RNAs, 23 transfer RNAs and 1 control region. The overall nucleotide composition is 27.92% for A, 24.66% for T, 30.90% for C and 16.52% for G. Among 23 tRNA genes, 8 tRNAs were encoded on the L-strand. Further, the phylogenetic tree, which based on complete mtDNA sequences, revealed that the *E. carlsbergi* was genetically closest to species *E. antarctica* and *Krefftichthys anderssoni.* This study could provide a basic data for the studies on evolution for low temperature adaptability, stock evaluation and conservation genetics.

In terms of biomass level, Electrona is the second largest marine organism in Antarctic Ocean and including three species of Electrona, like *Electrona antarctica*, *Electrona carlsbergi* and *Electrona rissoi* (Zhu et al. 2012). *E. carlsbergi* (Myctophidae, Electrona), appeared in the late Miocene, is one of the most abundant species in Antarctic waters. They cover waters from the south of the Antarctic convergence to the Antarctic coast and generally live in the depth of 200–400 m of water. The optimum water temperature is 1°C (Cheung et al. 2013). As an important member of the Antarctic ecosystem, *E. carlsbergi* mainly preys on *Antarctic krill*, *copepods*, and *cephalopods*. The distribution of *E. carlsbergi* is most affected by temperature. Their maximum length is 10.5 cm and the body of females are larger than males. So far, no complete mitochondrial sequence information is available. The study is important for the Antarctic ecosystem and further research on genetics and evolution of *E. carlsbergi*.

Adult fish of *E. carlsbergi* was collected from Antarctic (63°14′54″S, 59°51′24″W), it was transported to East China Sea Fisheries Research Institute, Chinese Academy of Fishery Science after freezing at −80°C. The genomic DNA was extracted from muscle tissues using Animal Genomic DNA Extraction Kit (TIANGEN) following the operation manual. The amplifying and sequencing primers were designed according to the sequence of *E. antarctica* (AP012248.1). We acquired the complete mitochondrial sequence of *E. carlsbergi* and submitted it into the Genbank database with an accession number MF596172. This complete mitochondrial genome is 18,282 bp in length, including 13 protein-coding genes, 2 rRNA genes, 23 tRNA genes and 1 control region. The overall nucleotide composition is 27.92% for A, 24.66% for T, 30.90% for C and 16.52% for G. The GC content (47.42%) is similar to *Chionodraco hamatus* (Song et al. 2016). In 13 protein-coding genes, there types of initiation codon (ATC, ATG, GTG) were identified. Four types of stop codons (TAA, TAG, TA, T) were detected and the rare stop codon TAG stopped ND3, it was different from *Gymnodraco acuticeps*. Eight of 23 tRNAs were encoded on the L-strand. The length of control region (D-loop) is 1990bp, longer than other fishes and its overall nucleotide composition is 35.23% for A, 31.81% for T, 21.61% for C and 11.36% for G. It has many long tandem repeat, like (ATTATACCCATAACTTGATATAACCC)_8_ and (ATATGTATTATACCC)_12_.

To evaluate the phylogenetic position of *E. carlsbergi* in Myctophidae fishes, the phylogenetic tree was reconstructed based on complete mtDNA sequences using the Neighbour-joining method in MEGA 5.1 (Kumar et al. 2008) (Figure 1). *Neoscopelus microchir*, *Neoscopelus macrolepidotus*, *Scopelengys tristis* and *Solivomer arenidens* were used as an out-group. The NJ tree showed that *E. carlsbergi* clustered with *E. antarctica* and *Krefftichthys anderssoni*, then together with other species in family Myctophidae, forming a big branch. Besides, the out-group in family Neoscopelidae formed a big sister branch as well.

**Figure 1.**
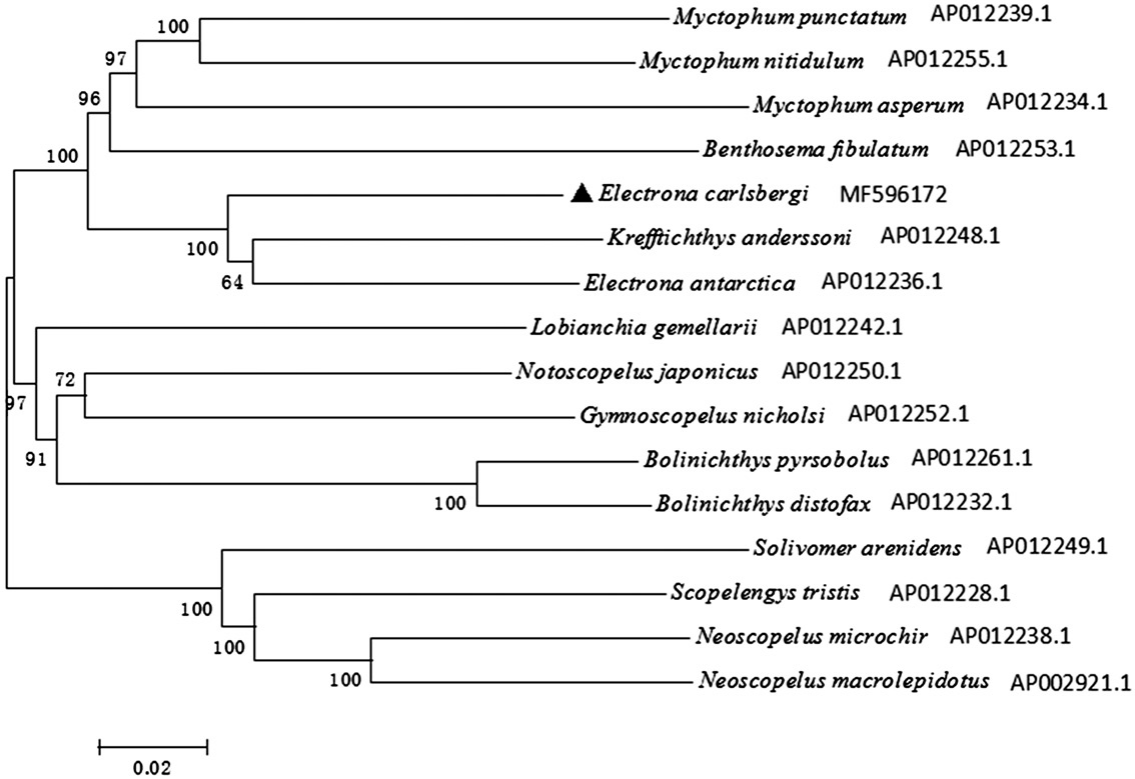
The phylogenetic tree based on complete mtDNA sequences using the Neighbour-joining method in MEGA 5.0. *Electrona carlsbergi* was highlighted with a black triangle.
